# anlotinib alters tumor immune microenvironment by downregulating PD-L1 expression on vascular endothelial cells

**DOI:** 10.1038/s41419-020-2511-3

**Published:** 2020-05-04

**Authors:** Shaochuan Liu, Tingting Qin, Zhujun Liu, Jing Wang, Yanan Jia, Yingfang Feng, Yuan Gao, Kai Li

**Affiliations:** 1 Tianjin Medical University Cancer Institute and Hospital, National Clinical Research Center for Cancer, Tianjin, China; 2 Key Laboratory of Cancer Prevention and Therapy, Tianjin, China; 3 Tianjin’s Clinical Research Center for Cancer, Tianjin, China; 4 Department of Thoracic Oncology, Tianjin Lung Cancer Center, Tianjin Cancer Institute & Hospital, Tianjin Medical University, Tianjin, 300060 China

**Keywords:** Immune evasion, Cancer microenvironment

## Abstract

Aberrant vascular network is a hallmark of cancer. However, the role of vascular endothelial cells (VECs)-expressing PD-L1 in tumor immune microenvironment and antiangiogenic therapy remains unclear. In this study, we used the specimens of cancer patients for immunohistochemical staining to observe the number of PD-L1^+^ CD34^+^ VECs and infiltrated immune cells inside tumor specimens. Immunofluorescence staining and flow cytometry were performed to observe the infiltration of CD8^+^ T cells and FoxP3^+^ T cells in tumor tissues. Here, we found that PD-L1 expression on VECs determined CD8^+^ T cells’, FoxP3^+^ T cells’ infiltration, and the prognosis of patients with lung adenocarcinoma. Anlotinib downregulated PD-L1 expression on VECs through the inactivation of AKT pathway, thereby improving the ratio of CD8/FoxP3 inside tumor and remolding the immune microenvironment. In conclusion, our results demonstrate that PD-L1 high expression on VECs inhibits the infiltration of CD8^+^ T cells, whereas promotes the aggregation of FoxP3^+^ T cells into tumor tissues, thus becoming an “immunosuppressive barrier”. Anlotinib can ameliorate the immuno-microenvironment by downregulating PD-L1 expression on VECs to inhibit tumor growth.

## Introduction

The aberrant vasculature, one of hallmarks of cancer, helps neoplasms to escape from immune attack in tumor microenvironment. In specific speaking, the abnormal vascular network will constitute an immunosuppressive environment and inhibit the infiltration of effector T cells including CD8^+^ cytotoxic T lymphocytes that are the key effectors of antitumor immunity^1,2^.

Furthermore, aberrant vascular endothelial cells (VECs) can also participate in immune inhibition and suppress the function of immune cells in tumor microenvironment by expressing inhibitory receptors such as FasL, VACM-1, PD-L1 (program death ligand 1) and CD73, etc^3,4^. PD-L1 is one of the ligands of PD-1 and wildly expressed on various cells (such as immune cells, non-hematopoietic cells and tumor cells, etc.) in the tumor microenvironment^5^. As a variety of targeted drugs against PD-1/PD-L1 have obtained good results in many clinical trials, the PD-1/PD-L1 pathway has become the hotspots of study. It has been confirmed that PD-L1 expression on VECs enhances the inhibitory function of FoxP3^+^ Treg cells^6^ and declines the function of CD8^+^ T cells in various autoimmune diseases^7,8^. However, it is still poorly understood about the detail of immune-modulatory effects of PD-L1 expression on VECs in tumor microenvironment.

According to the previous literature^9^, proinflammatory signaling may facilitate PD-L1 expression on VECs and decrease the efficacy of immunotherapies. Some studies^10,11^ suggest that the expression of PD-L1 on VECs is upregulated by increased IFN-γ secreted after antiangiogenic therapy. According to these studies, we reasonably speculate that the expression of PD-L1 on VECs will participate in antiangiogenic therapy and even affect the efficacy.

Anlotinib, a novel orally administered RTK inhibitor^12^, has showed promising efficacy in several clinical trials via inhibiting activation of various pro-angiogenic signals (VEGFR, PDGFR, and FGFR, etc.)^13,14^. However, the effect of anlotinib in tumor immune microenvironment remains largely unclear. Thus, this study aims to investigate the effects and underlying mechanism of anlotinib on PD-L1 expression in tumor VECs and tumor immune microenvironment.

We have demonstrated in this study that anlotinib can inhibit PD-L1 expression on VECs so as to break through the “immune tolerance barrier”, promote CD8^+^ T cells infiltration, improve the balance of CD8/FoxP3. This work provides new insights into the effects of antiangiogenic therapy on tumor immune microenvironment, which is mediated by the change in the expression of PD-L1 on VECs. In addition, this work reveals a new evidence and proposes a possible mechanism by which anlotinib affects the immune microenvironment to inhibit tumor cell proliferation.

## Materials and methods

### Cell culture and reagents

Human umbilical vein endothelial cells (HUVECs) and A549 were obtained from Peking union medical college cell bank; immortalized mouse brain endothelial cell lines (bEnd.3) were obtained from Nankai University; mouse retinal microvascular endothelial cells (MRMECs) were gifts from Tianjin medical university eye hospital; MC38 cells, B16 cells and B16-OVA cells were obtained from Dr. Yuan at Tianjin Medical University Cancer Institute and Hospital. All cell lines were performed cell line authentication before receipt, and all of the cell lines were passaged for no >6 months. Hypoxia (<1% O_2_) was induced by a modular incubator chamber (Billups-Rothenberg). Bevacizumab (H0180801, Roche) was purchased from Roche company. Anlotinib and anti-PD-L1 drug were gifts from Nanjing Chia Tai Tianqing Company. Endothelial cells and B16 cells were grown using DMEM medium (Lonza) containing 10% fetal bovine serum (FBS). MC38 cells were grown with RMPI 1640 (Thermo Flasher scientific) containing 10% FBS. All cells were starved for 12 h before being stimulated by cytokines or the supernatants of tumor cells.

### Western blots analysis

HUVECs, bEnd.3, and MEMRC were stimulated by the supernatant medium of A549 cells, vascular endothelial cell growth factor A (VEGFA) or cultured under hypoxia condition etc. for 24 h. Protein samples were collected in ice-cold 1× RIPA buffer containing a protease inhibitor cocktail (Thermo Flasher scientific) and were separated using 10% sodium dodecyl sulphate-polyacrylamide gel electrophoresis. The proteins were transferred to polyvinylidenedifluoride membranes (Roche Molecular Biochemicals, Quebec, Canada), blocked with 5% bovine serum albumin for 1 h, and then incubated overnight at 4°C with appropriate primary antibodies. We used the primary antibodies including: PD-L1 (ab213524, Abcam), AKT (4691, Cell Signaling Technology), p-AKT (4060, Cell Signaling Technology), ERK (4695, Cell Signaling Technology), p-ERK (4370, Cell Signaling Technology), β-actin (8456, Cell Signaling Technology), PI3k (4255, Cell Signaling Technology), p-PI3K (4228, Cell Signaling Technology). The blots were further incubated with conjugated secondary antibodies and washed 3 times in TBST for 5 minutes each time. The blots were further incubated with HRP-conjugated secondary antibodies and developed with the ECL System (Millipore).

### Endothelial cells–T cells co-cultures

HUVECs were pre-incubated with the supernatant conditioned medium (CM) of A549 cells for 48 h before co-culturing with PBMC. PBMC were extracted from normal blood of healthy volunteers and treated with 2.5 µg/ml anti-CD3 (OKT3, eBioscience) antibody and IL-2 (100 U/ml) in order to stimulate the activation of T cells. After 72 h of T-cell activation, PBMC were incubated with A549 CM-treated HUVEC cells at a 1:1 ratio for 48 h. Different inhibitors or drugs, such as anti-PD-L1 drugs (10 µg/ml), anti-VEGF antibody (10 µg/ml), and anlotinib (1 µm) were added to the mix. The proliferation of T cells was measured by flow cytometric measurement of Ki67.

### Paraffin section of human tumor

All paraffin sections of patient’s tumor tissues were obtained from Tianjin Medical University Cancer Institute and Hospital. We collected all sections from patients with colon (*n* = 50), renal (*n* = 32) and lung cancers (primary and metastatic tumor samples from 106 patients with lung adenocarcinoma with treatment-naive stage I–IV) for immunohistochemical staining. All patients underwent surgery or treatment at Tianjin Medical University Cancer Institute and Hospital between 2011 and 2018. The clinical data of the patients collected included: age at diagnosis, gender, smoking history, gene mutation status, and clinical stage. All clinical parameters were retrieved according to the electronic medical record. The study was approved by the Tianjin Medical University Cancer Institute and Hospital’s Ethics Committee and informed consents were obtained from all patients.

### Quantitation of TILs (tumor-infiltrating lymphocytes)

Paraffin-embedded patient tumor tissue sections were placed at 70°C for 1 h or overnight, and immersed in xylene to remove paraffin, rehydrated in a continuous alcohol grading and washed in water for 5 min. Sections were placed in citrate or ethylenediaminetetraacetic acid (depending on the antibody), and antigen retrieval were performed at 130°C for 2 min. After being blocked with 3% hydrogen peroxide and normal goat serum, the sections were incubated with primary antibody at 4°C overnight, followed by EIVISON plus (kit-9903, MXB), DAB kit (ZL1-9019, ZSGB-BIO) per the manufacturers protocol were used for coloration. Finally, the tumor sections were stained with hematoxylin. All stained slides were scanned under high magnification (×20 objective lens), and four non-overlapping areas (excluding necrotic areas) were selected from each tumor tissue, which was rich in FoxP3^+^ (ab20034, Abcam) or CD8^+^ (ab9328, Abcam) TILs. The final number of TILs for each section was based on the average of the four fields. The number of TILs was counted using Image J software (http://rsb.info.nih.gov/ij/).

### Evaluation of the percent of PD-L1^+^ blood vessels and the positive rate of VEGFA and HIF-1α in tumor

For evaluation of the percent of CD34^+^ PD-L1^+^ vessels in tumor tissues. All patient sections were double-stained with anti-CD34 antibody (ZA-0550, ZSGB-BIO) and anti-PD-L1 antibody (66248-1-lg, Proteintech) using a double-staining kit (DS-0001, ZSGB-BIO). First, the most-abundant sites of blood vessels were selected under fourfold magnification of microscope, and four sites were randomly selected under a 40-fold objective lens, and the proportion of PD-L1^+^ CD34^+^ cells in all CD34^+^ vessels was calculated and averaged. The average percentage was used as the final result for each section. To evaluate the positive expression of VEGFA (ab1316, Abcam) and hypoxia-inducible factor α (HIF-1α) (ab113642, Abcam), stained sections were digitally analyzed at ×400 resolution using an Olympus BX-UCB. HIF-1α and VEGFA expression were scored by the H-score system, respectively. *H* score calculation method is based on previous literature^15^.

### Flow cytometry

Preparation of single-cell suspension of mouse tumor tissues by mechanical grinding. All single-cell suspensions were incubated with rat anti-mouse CD16/CD32 blocking antibody (4 μg/ml) for 15 min after thorough filtration and precipitation, stained with fluorescein-conjugated antibodies, multiple washed with PBS, and resuspended in 7-AAD (exclude non-viable cells). For anti-mouse CD4 (Clone RM4-5, BD Biosciences), anti-mouse CD8 (clone 53-6.7, eBioscience), anti-mouse CD45 (clone 30-F11, Biolegend), anti-mouse CD25 (PC61.5, eBioscience), anti-mouse PD-L1 (clone 10 F.9G2, BioLegend) and anti-mouse CD31 (clone MEC 13.3, BD Biosciences) staining, after incubation for 1 h, cells were washed with PBS for three times (1500 rpm, 5 min each), then detected by flow cytometry (BD FACSCanto II). For FoxP3 staining, after incubation, cells were washed and fixed with 1 ml of fixation & permeabilization solution (BD Biosciences) for 30–60 min, and washed twice with Perm Wash (BD Biosciences). Intracellular staining with anti-FoxP3 antibody (clone FJK-16s, eBioscience) was performed for 1 h. The next steps are the same as described above.

### Immunofluorescence

Fresh frozen tumor sections (stored at −80°C) were fixed in precooled 4% paraformaldehyde for 15 min at room temperature. The fixed frozen samples were permeabilized with 0.2% Triton X-100 (Applichem) in PBS for 10 minutes. After washing with PBS for three times, 5 min per time, all samples were incubated with blocking solution containing 1% BSA (Sigma), 0.01% Triton X-100 and 10% FBS in PBS for 1 h. Next, the sections were incubated with primary antibody at 4°C overnight. Primary antibodies used were as follows: FITC-CD31 (clone MEC 13.3, BioLegend), rabbit anti-mouse CD31 (ab28364, Abcam), APC-PD-L1 (clone 10 F.9G2, BioLegend), rabbit anti-mouse PD-L1 (LS‑C746930, LifeSpan BioSciences), rat anti-mouse CD4 (Clone RM4-5, BD Biosciences), rat anti-mouse CD8 (clone 53-6.7, eBioscience) and rat anti-mouse FoxP3 (clone FJK-16s, eBioscience). Then, the sections were rewarmed at room temperature for 15 min, followed by washing three times in PBS for 5 min per time. At last, tissue sections were stained with secondary antibodies and incubated for 1 h at room temperature. The secondary antibodies used included: donkey anti-goat AF488 (SA5-10086, Invitrogen); donkey anti-rat AF488 (A-21208, Invitrogen); donkey anti-goat AF568 (A-11057, Invitrogen); donkey anti-rabbit AF647 (A-31573, Invitrogen); washed three times again in PBS, and subsequently stained with anti-fluorescence quencher (including DAPI). All stained sections were stored at −20°C and used for image acquisition using Zeiss Imaginer-Z2. According to previous study^10^, if CD4^+^, CD8^+^, or FoxP3^+^ T cells are contained within a 25-μm radius from the CD31^+^ vascular structure, it is defined as “perivascular immune cells”.

### In vivo experiment

C57BL/6 J female mice (6 weeks) were purchased from the Model Animal Center of Nanjing University. All experimental procedures were in accordance with the protocols approved by the Institutional Animal Care and Research Advisory Committee of Tianjin Medical University. In order to construct tumor-bearing mouse models of B16 or MC38 cells, 1 × 10^6^/100 μl cancer cells were injected subcutaneously into each mouse. B16 and MC38 tumors were grown for several weeks and observed every other day. From the 11th day, mice were randomly divided into PBS, anti-VEGF (bevacizumab, 10 mg/kg, every 3 days), anlotinib (1.5 mg/kg, every day) and combination groups, each group was given different treatment, intraperitoneal injection of anti-VEGF or gavage anlotinib. According to the previous study^10^, The calculation formula of tumor volume is *V* = *π* × width × width × length/6. We set the B16 tumors reached a mean volume of 2000 mm^3^ as the end time of mice’ survival and set the MC38 tumors reached a mean volume of 1000 mm^3^ as the end time of mice’ survival.

### T-cell depletion

In T-cell depletion experiments, we used antibodies included CD4 depletion antibody (clone GK1.5, BioXcell) and CD8 depletion antibody (clone 53-6.7, BioXcell). For 1 day immediately before treatment with anlotinib, mice were separately treated i.p. twice weekly with 200 µg of the CD8 depletion antibody, CD4 depletion antibody and combination of both in different groups. Depletion efficiency was determined by flow cytometric analysis of mice spleen tissue.

### bEnd.3 model

Mouse endothelial cells of bEnd.3 were transduced with a lentiviral expression vector (overexpressed CD274 (PD-L1)). In all, 1 × 10^6^ empty vector or CD274-transduced bEnd.3 cells were injected intratumorally (i.t.) into B16 tumors on day13, followed by an additional i.t. injection at next week. Treatments with anlotinib were carried out as described for the above experiments before 1 day injection with bEnd,3 cells.

### Statistics

All Statistical analyses were performed using SPSS v.21 (IBM Corp), and all statistical graphs and survival curves were generated using GraphPad Prism 6 (USA, GraphPad Software). Patients’ overall survival (OS) and progression-free survival (PFS) were estimated by the Kaplan–Meier method and compared using the log rank’s test. One-way analysis of variance was performed for tumor growth analysis. Differences between the two groups were calculated using an unpaired Student’s *t* test. *P* < 0.05 was considered significant statistical differences.

## Results

### The VECs in different human cancers express PD-L1

**Fig. 1:**
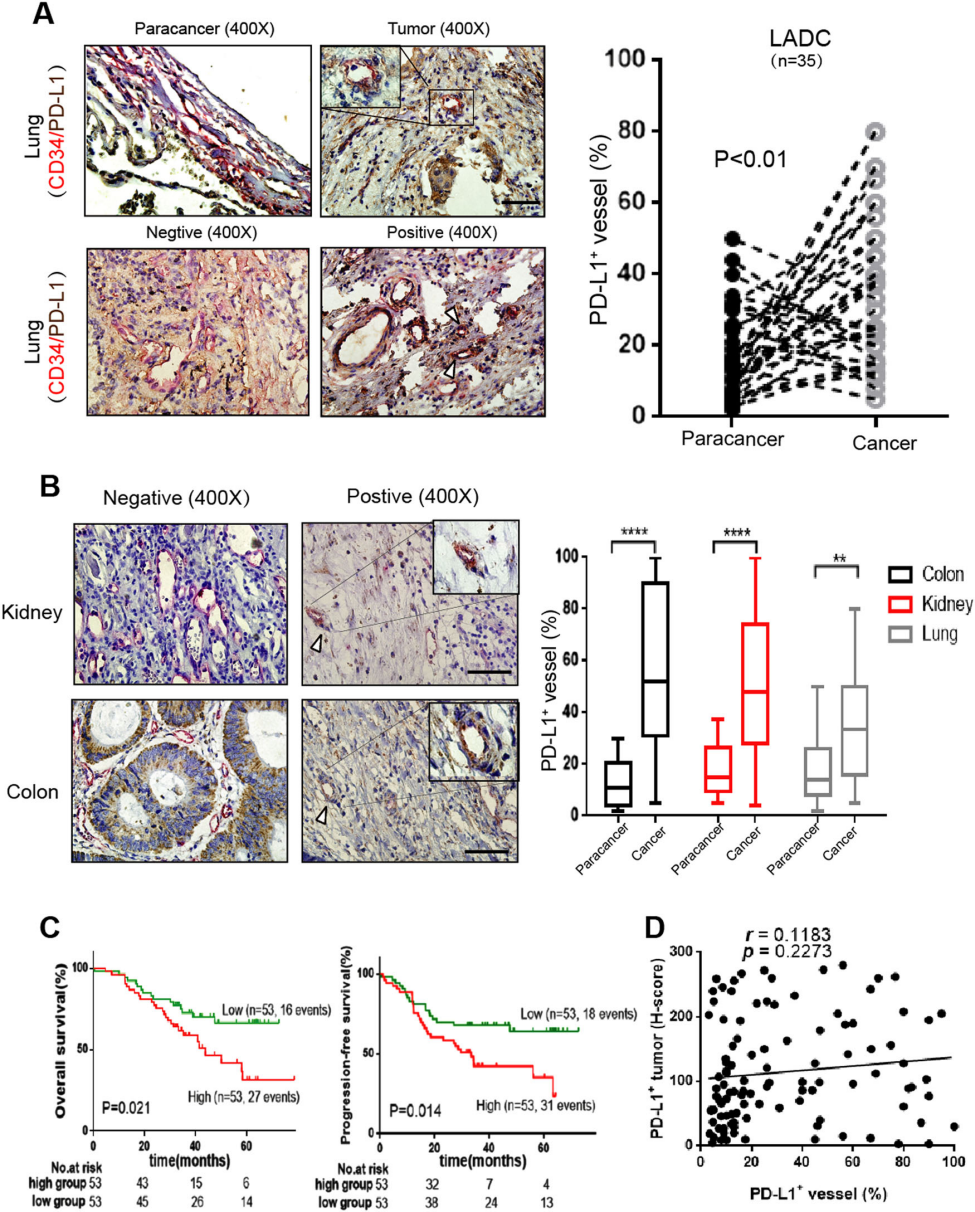
PD-L1 high expression on tumor VECs is closely related to the prognosis of patients. **a**, **b** Typical images of CD34/PD-L1 (endothelial cells highly expressing PD-L1) immunostaining in lung adenocarcinoma (*n* = 35), kidney (*n* = 30), and colon cancer (*n* = 30), scale bars: 50 μm. Kaplan–Meier survival curves of OS **c** and PFS **c** of patients with lung adenocarcinoma exhibiting high (red) or low (green) VEC-PD-L1. **d** The correlation of PD-L1^+^ vessels and PD-L1^+^ tumor tissues (*n* = 106). Data are mean ± SD, *P < 0.05, **P < 0.01, ***P < 0.001, ****P < 0.0001, Student’s *t* test.

Although it has been reported in the literature that PD-L1 is expressed on the VECs, the status of PD-L1 expression on VECs inside tumor tissues has not been detailed reported. We analyzed the PD-L1 expression in various samples of human cancers including lung adenocarcinoma (*n* = 35), colon cancer (*n* = 50), renal cancer (*n* = 32), and the corresponding para-carcinoma tissues. Here we found that PD-L1 expression in the normal lung tissue was low or none, whereas a significant percentage of CD34^+^ blood vessels exhibited high PD-L1 expression in lung adenocarcinoma tissue, colon cancer and renal cancer (Fig. 1a, b). Moreover, we observed that the high expression of PD-L1 on VECs predicted a poor PFS (*P* = 0.014) and OS (*P* = 0.021). As for the relationship between the expression of PD-L1 and the characteristics of patients with lung adenocarcinoma (*n* = 106), we did not find a significant correlation between the percentage of CD34^+^ PD-L1^+^ vessels and any clinical variables, even though *P* value is closed to significant point (0.066) in T stage (Fig. 1c and Table S1). Though in previous studies^16,17^, the tumor-expressing PD-L1 was always a poor prognostic factor for lung adenocarcinoma, we also did not find a close relation between the PD-L1 expressed in tumor (T-PD-L1) and VECs (VEC-PD-L1) (*r* = 0.118 *p* = 0.227) (Fig. 1d). This result indicates that the expression of PD-L1 on VECs does not depend on the expression of PD-L1 on tumor cells, but affects independently the prognosis of lung adenocarcinoma.

### PD-L1 expressed on human VECs participates in immune regulation

**Fig. 2:**
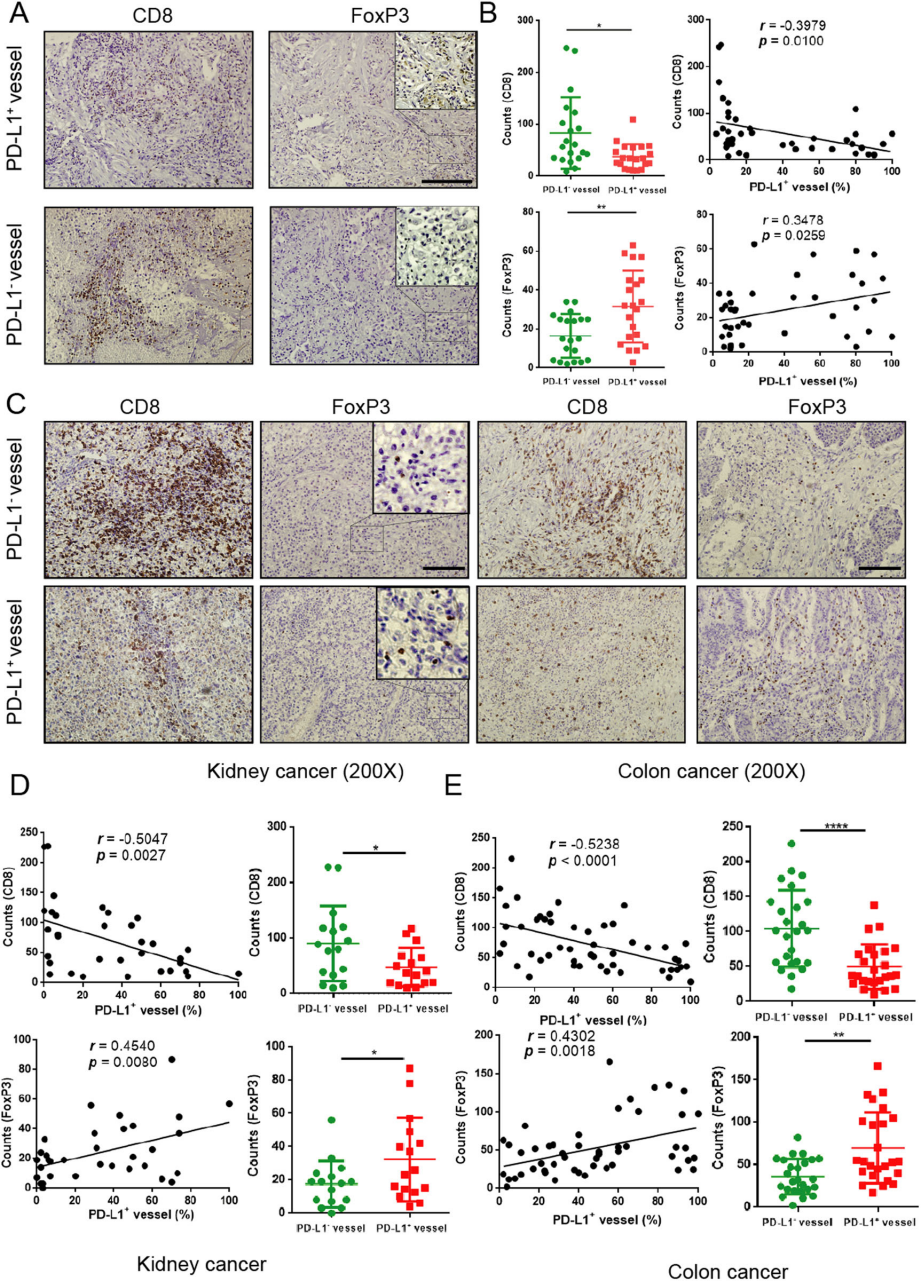
PD-L1 high expression on VECs is closely related to the infiltration of immune cells. **a**, **c** Representative images of CD8^+^T cells and FoxP3^+^ T cells in sections taken from subjects with either PD-L1^+^ or PD-L1^−^ vessels in lung, kidney, and colon cancer. Original magnification, ×200. The number of intratumoral CD8^+^ T cells and FoxP3^+^ T cells from patients’ sections (kidney or colon cancer) associated with the percentage of PD-L1^+^ vessels. The determination as high and low were grouped by the median values. Lung adenocarcinoma (*n* = 41) **b**, colon cancer (*n* = 50) **e** and kidney cancer (*n* = 32) **d**; scale bars: 100 µm. Data are mean ± SD. *P < 0.05, **P < 0.01, ***P < 0.001, ****P < 0.0001, Student’s *t* test.

As a recognized co-inhibitory molecular for T cells, PD-L1 can bind to PD-1 to inhibit the biological activity of T lymphocytes, thereby inhibiting T-cell-mediated tumor-specific immunity. According to previous literature^18,19^, the immune microenvironment in tumor tissues is closely related to the balance of CD8^+^ T cells and FoxP3^+^ Treg cells. In three vasculature-rich tumors (lung, colon, and kidney cancers), we found that VECs with overexpression of PD-L1 were concomitantly with higher infiltration of FoxP3^+^ Treg cells (*r* = 0.348, *P* = 0.026), but with lower infiltration of CD8^+^ T cells (*r* = −0.398, *P* = 0.010) in lung adenocarcinomas (Fig. 2a, b). In order to verify this result, we extended to additional tumors including specimens of colon and kidney cancer, and found that overexpression of PD-L1 on VECs was also associated with fewer CD8^+^ T cells and more FoxP3^+^ Treg cells in these two cancer tissues (Fig. 2c–e). These findings indicate that the upregulation of PD-L1 expression on tumor VECs may inhibit the infiltration of CD8^+^ T cells and activate FoxP3^+^ Treg cells.

### PD-L1 expression on VECs affects the proliferation and activation of immune cells

**Fig. 3:**
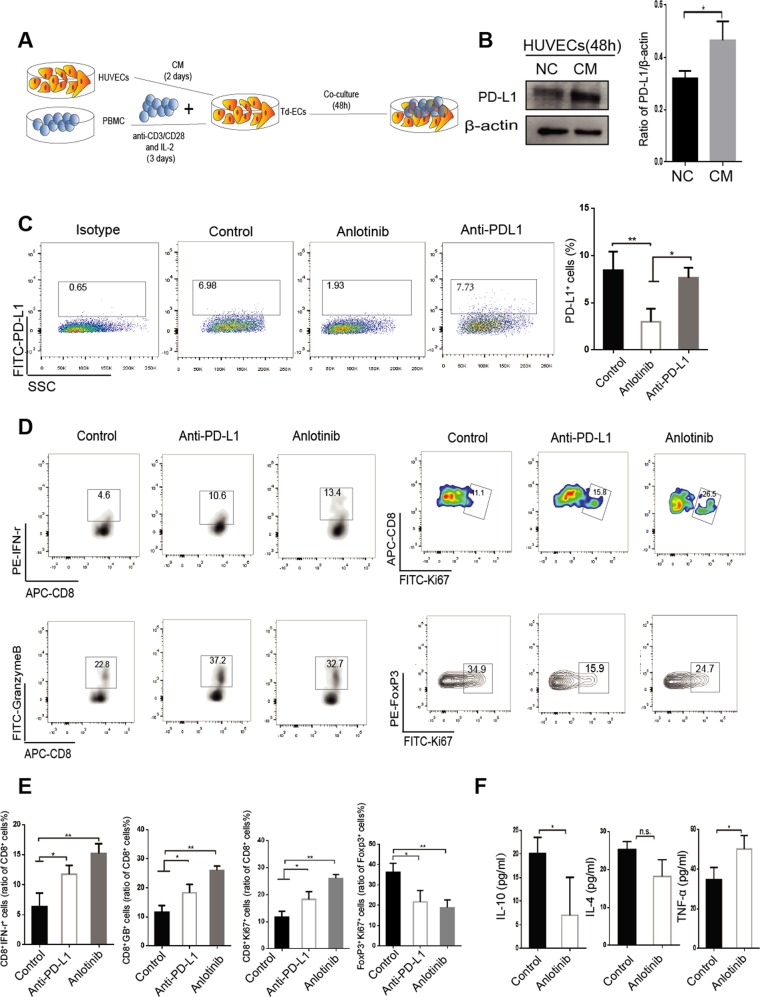
PD-L1 expression on endothelial cells affects the proliferation and activation of immune cells. **a** PBMC was extracted from normal blood and treated with 2.5 µg/ml anti-CD3 antibody and IL-2 (100 U/ml) for 72 h, and co-cultured with tumor-derived endothelial cells (Td-EC) for 48 h. **b** HUVECs were treated with the supernatant of tumor cells (CM) for 48 h. **c** PBMC and Td-EC were treated with control, anlotinib (0.1 μm) or anti-PD-L1 antibody (4 μg/ml). **d** Detection of the CD8^+^ IFN-γ^+^, CD8^+^Ki67^+^, CD8^+^GranzymeB^+^, and FoxP3^+^ T cells by flow cytometry. **e** Quantification of the above positive cells in the immune cells. **f** Quantification of cytokine levels in co-culture supernatants (2 days) by liquid microarray. Data are mean ± SD. *P < 0.05, **P < 0.01, ***P < 0.001, Student’s *t* test.

Monocytes were extracted from the peripheral blood of healthy volunteers with complete consent, activated in vitro with anti-CD3 and IL-2 for 72 h, and co-cultured with VECs treated by the supernatant of A549 cells for 48 h (Fig. 3a). We found that CM of tumor could significantly increase PD-L1 expression on VECs at 48 h (Fig. 3b). In addition, PD-L1 expression on VECs could be inhibited by treatment with anlotinib (Fig. 3c). After VEC-PD-L1 inhibition (treated with Anlotinb or anti-PD-L1 monoclonal antibody), we observed an enhancement in the proliferation capacity of CD8^+^ T cells and the activation of CD8^+^ T cells (increased secretion of IFN-γ and granzyme B) (Fig. 3d, e). Furthermore, anlotinib or anti-PD-L1 drug could effectively decrease the proliferation of FoxP3^+^ T cells (Fig. 3d, e). Through using a liquid chip to detect IL-4, IL-10, and TNF-α in the supernatant of co-culture, we found that anlotinib significantly inhibited the secretion of IL-10 and IL-4 and promoted the secretion of TNF-α (Fig. 3f). These results suggest that VEC-PD-L1 can affect the proliferation and activation of CD8^+^ T cells, as well as Treg cells.

### Anlotinib inhibits PD-L1 expression upregulated by VEGFA and HIF-1α via AKT signals on VECs

By staining paraffin sections of lung adenocarcinoma, we found that the VECs with PD-L1 overexpression simultaneously revealed higher expression of VEGFA, HIF-1α, and microvascular density compared with negative group (Fig. S1a, b). This result suggests that the number of blood vessels, angiogenic growth factors, and HIF expression in tumor tissues are probably closely related to VEC-PD-L1.

In agreement with the results of our experiment in vitro, PD-L1 expression on VECs was facilitated under conditions of CM of tumor, hypoxia or FBS, but this phenomenon was not observed only with VEGFA stimulation (Fig. S1c). It suggests that VEGFA may not be the initiator for the PD-L1 expression on VECs alone and multiple factors, probably including HIF-1α, could drive PD-L1 expression. It has been reported in one literature that antiangiogenic drugs can block pro-angiogenic factors and improve hypoxia in tumor to a certain extent^20^. As a novel small molecule tyrosinase inhibitor, anlotinib can effectively inhibit angiogenesis and improve the perfusion of tumor tissues.

**Fig. 4:**
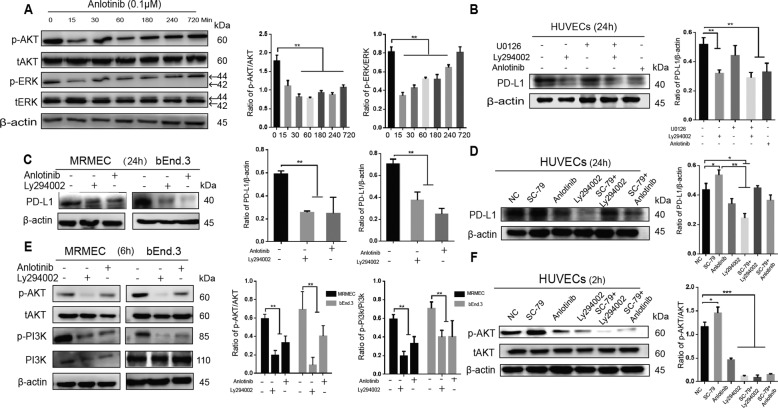
anlotinib can inhibit endothelial PD-L1 expression via the AKT pathway. **a** Western blot analysis showed p-AKT and p-ERK levels in with- or without-anlotinib (0.1 µm) treated HUVECs at the indicated time points. **b** Western blot analysis showed PD-L1 expression levels in HUVECs treated by U0126 (ERK inhibitor,10 µm), Ly294002 (AKT inhibitor, 10 µm), or anlotinib (0.1 µm) at 24 h. **c**, **e** Western blot analysis showed p-AKT and PD-L1 expression in MRMEC and bEnd.3 treated as shown. **d**, **f** Western blot analysis showed p-AKT and PD-L1 expression in HUVECs treated as shown. (SC-79, one of activator of AKT). Data are mean ± SD. *P < 0.05, **P < 0.01, ***P < 0.001, ****P < 0.0001, Student’s *t* test.

Based on the results of western blot, we found that anlotinib inhibited PD-L1 expression on VECs in hypoxia or in CM of tumor (Fig. S1d). In addition, anltotinib inhibited the activation of the PI3K/AKT pathway (Fig. 4a). Therefore, we blocked the AKT pathway, then found the inhibition of PD-L1 expression on HUVECs (Fig. 4b), and the same event was observed in the MRMEC and bEnd.3 cells (Fig. 4c and e). Moreover, we found that PD-L1 expression was increased after the addition of AKT activator (SC-79), whereas it was inhibited again after the addition of AKT inhibitor (LY294002) (Fig. 4d and f). It indicates that the AKT pathway is involved in the regulation of PD-L1 in endothelial cells. In order to further investigate the mechanism of anlotinib’s effect on endothelial PD-L1 expression, we selected the VEGFR phosphorylation inhibitor SU5408 (SU), the PDGFR phosphorylation inhibitor CP-673451 (CP) and the FGFR phosphorylation inhibitor FIIN-2 (FI) to block a variety of pro-angiogenic receptors. We found that the blockage of phosphorylation of VEGFR, PDGFR, or FGFR could downregulate the PD-L1 expression on VECs via inhibiting the activation of the AKT pathway (Fig. S1e).

### Anlotinib inhibits PD-L1 expression on VECs, improves the immune microenvironment via increasing the ratio of CD8/FoxP3 and suppresses tumor growth

To evaluate the impact of tumor-endothelial PD-L1 on the infiltration of CD8^+^ T cells and FoxP3^+^ T cells, we selected two xenograft animal models (MC38 and B16 cell lines). According to previous studies^21,22^, inhibition of PD-L1 expression in stromal cells is essential for antitumor immunity of B16 melanoma. On the contrary, the PD-L1 expression in MC38 colorectal adenocarcinoma cells is sufficient to suppress antitumor immunity. Therefore, B16 xenograft model was selected as the observation to better analyze the impact of PD-L1 expression on tumor VECs.

According to previous study^10^, anti-VEGF therapy upregulates PD-L1 expression on VECs in response to increased IFN-γ secreted by the perivascular CD8^+^ T cells. In our study, we found the similar phenomenon that the blockade of VEGF (bevacizumab) promoted VEC-PD-L1 in vitro experiment (Fig. S1f). In addition, anlotinib inhibited endothelial PD-L1 expression induced by bevacizumab (Fig. S1f). Therefore, we selected bevacizumab as a positive control group to observe the endothelial PD-L1 expression and the infiltration of immune cells in tumor tissues when angiogenesis was inhibited. In addition, we found that both bevacizumab and anlotinib had the similar angiogenesis inhibitory effects (Fig. S2A).

**Fig. 5:**
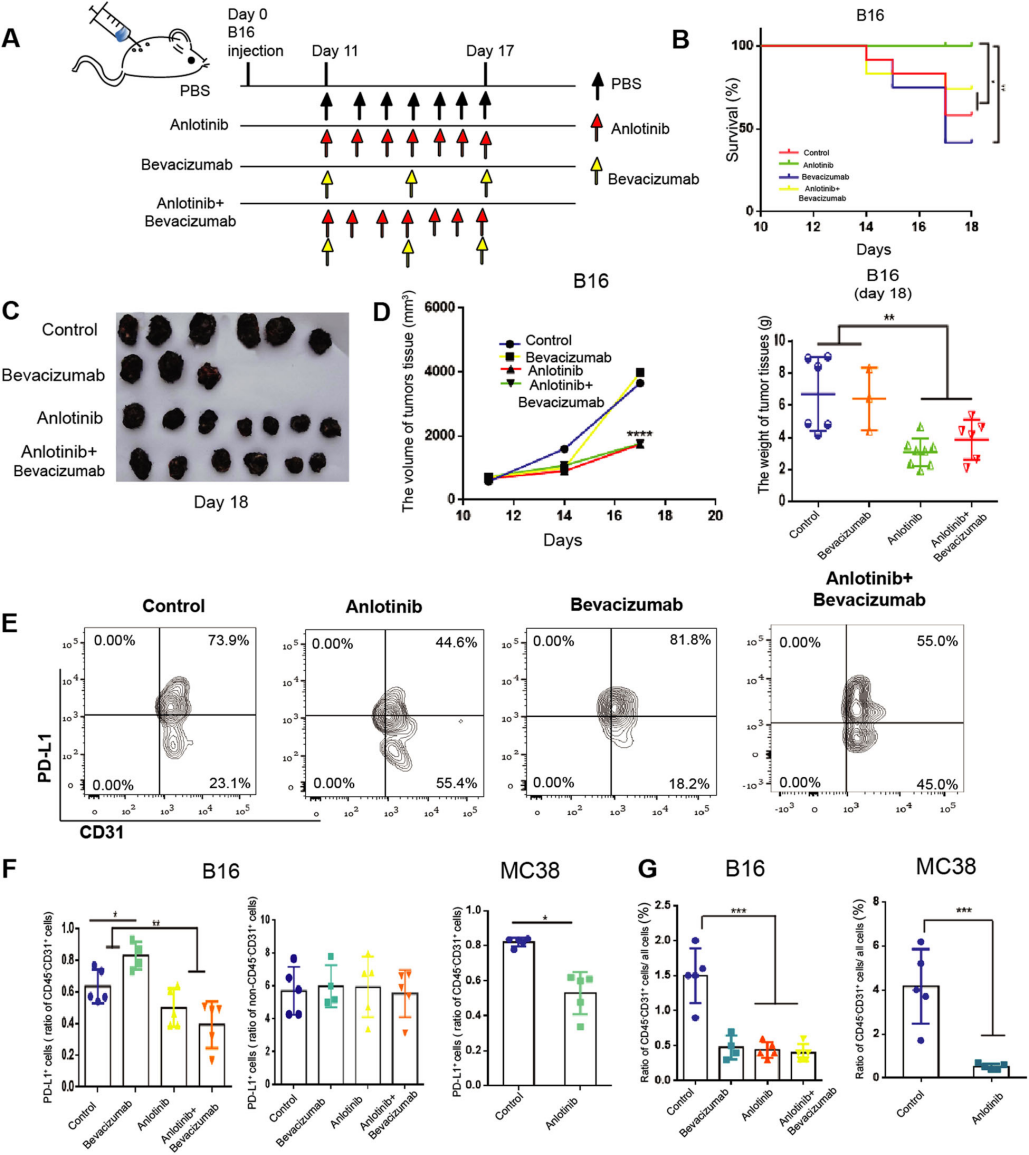
anlotinib inhibits endothelial PD-L1 expression and inhibits tumor growth. **a** C57BL/6 mice were injected with 1 × 10^6^ B16 cells and tumors grew. On day 11, mice were divided into four groups (*n* = 10 per group), and treated with bevacizumab, anlotinib, or both as shown. **b** Kaplan–Meier survival curves of B16 mice treated as indicated. **c** Representative images of B16 tumors in different groups. **d** Left: tumor growth curve of the various treatment groups; right: statistics of the weight of B16 tumors treated as indicated. **e** Representative flow images of CD31^+^ PD-L1^+^ cells taken from mice treated with anlotinib (*n* = 5), bevacizumab (*n* = 4), or both (*n* = 5). **f** Statistics of the percentage of CD31^+^ PD-L1^+^ cells in the B16 or MC38 tumors and the percentage of PD-L1^+^ non -VECs in B16 tumors. **g** Statistics of the percentage of CD45^−^CD31^+^ cells in the B16 or MC38 tumors. Data are mean ± SD. *P < 0.05, **P < 0.01, ***P < 0.001, ****P < 0.0001, Student’s *t* test.

In the B16 xenograft model, we found that anlotinib reduced the mortality in mice and decreased the volume and weight of tumors (Fig. 5a–d). In the MC38 group, anlotinib also decreased the size of the transplanted tumors (Fig. S2b). However, treatment with bevacizumab increased the mortality, tumor volume, and tumor weight of mice. When combined with anlotinib during bevacizumab treatment, it significantly decreased the mortality and tumor volume of mice compared with the bevacizumab alone group (Fig. 5a–d). Based on the results in vitro experiments, we speculated that the different result of treatment might be closely related to endothelial PD-L1 expression. Therefore, we found that anlotinib and combined group significantly inhibited VEC-PD-L1, whereas bevacizumab strongly upregulated VEC-PD-L1 in tumor-bearing mice (Fig. 5e–g). In order to further study the status of PD-L1 expression on tumor cells, we used flow cytometry and immunofluorescence (IF) staining and found that neither anlotinib, bevacizumab or the combination of them had any significant effects on PD-L1 expression in non-VECs in tumor tissues, nor on PD-L1 expression in tumor cells (Fig. 5f and Fig. S2c). These results again suggest that VEC-PD-L1 is closely related to the immune microenvironment of tumor tissues and the efficacy of antiangiogenesis therapy.

**Fig. 6:**
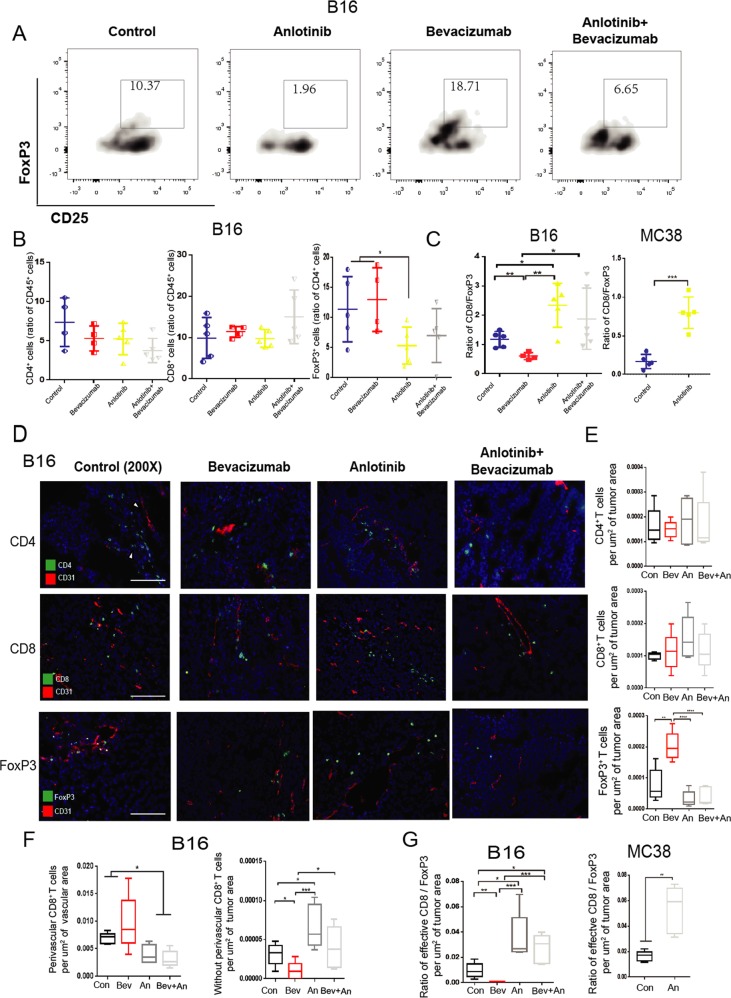
anlotinib improves the immune microenvironment via increasing the ratio of CD8/FoxP3. **a** Representative flow images of CD4^+^ CD25^+^ FoxP3^+^ T cells taken from B16 tumors treated as above indicated. **b**, **c** Statistics of the percentage of CD4^+^T cells, CD8^+^ T cells, FoxP3^+^ T cells and ratio of CD8/FoxP3 in the B16 or MC38 tumors. **d** Representative images of CD31 immunostaining (red), 4′,6-diamidino-2-phenylindole (DAPI) nuclear staining (blue) and markers of immune cells (CD4, CD8, and FoxP3) (green) of B16 tumors (*n* = 5 per group, except for Bev group *n* = 4) treated as above indicated. Scale bars,100 mm. **e** Quantification of CD4^+^ T cells, CD8^+^ T cells, FoxP3^+^ T cells in B16 tumors. **f** Statistics of perivascular and without perivascular CD8^+^ T cells in the B16 tumors. **g** Statistics of ratio of CD8/FoxP3 in the B16 or MC38 tumors. All data are exhibited as mean ± SD. Statistical differences were assessed using the unpaired Student’s test. *P < 0.05, **P < 0.01, ***P < 0.001, ****P < 0.0001.

In further analysis of the infiltration of immune cells, we found that compared with the control group, anlotinib and combination treatment group significantly inhibited the aggregation of CD45^+^ CD4^+^ CD25^+^ FoxP3^+^ T cells in the tumor tissues (Fig. 6a, b). However, treatment with bevacizumab did not inhibit or even slightly increase the infiltration of FoxP3^+^ T cells break through in the B16 xenograft model, and had a lower the ratio of CD8/FoxP3 compared with other groups (Fig. 6a, b). However, anlotinib group had a significant higher the ratio of CD8/FoxP3 (Fig. 6c). In the MC38 xenograft model, we found the similar phenomenon that anlotinib promoted the ratio of CD8/FoxP3 compared with the control group (Fig. 6c).

In addition, IF was used to analyze the degree of infiltration of immune cells in tumor tissues, and we found that there was no significant difference in the infiltration number of CD4^+^ T cells between different groups (Fig. 6d, e). Although we did not find a significant increase in the infiltration number of CD8^+^ T cells in anlotinib group, there was a conspicuous upward trend compared with the control group (Fig. 6d, e). Moreover, it is considered that the CD8^+^ T cells around blood vessels as “inefficacious” immune cells can not to penetrate the immune barrier and infiltrate into tumor tissue. By counting we found a large amount of CD8^+^ T cells clustered around the blood vessels in the bevacizumab group, while infiltrated CD8^+^ T cells were much lower than those of the anlotinib or combination treatment group (Fig. 6f). In addition, we found the infiltration number of FoxP3^+^ T cells was significantly reduced in anlotinib group, thereby improving the ratio of CD8/FoxP3 (Fig. 6e–g). On the other hand, bevacizumab group had a lowest ratio of CD8/FoxP3 compared with the other groups, which exacerbated the immunosuppressive microenvironment (Fig. 6f, g). In addition, in the MC38 xenograft model, we observed that anlotinib group could also significantly improve the ratio of CD8/FoxP3 (Fig. 6g). These results suggest that anlotinib can break through the immunosuppressive barrier of VEC-PD-L1 and significantly increase the ratio of CD8/FoxP3 in tumor tissues.

### PD-L1 expressed on VECs affects the efficacy of anlotinib

To determine the effect of immune function of anlotinib treatment, we used CD4 and CD8 depletion antibodies to deplete CD4^+^ T and CD8^+^ T cells. We found that the efficacy of anlotinib was significantly reduced after depletion of CD8^+^ T cells, whereas depletion of CD4^+^ T cells alone had no significant effect on anlotinib’s efficacy (Fig. S3a–c). These results indicate that the function of CD8^+^ T cells has an important role in treatment with anlotinib.

**Fig. 7:**
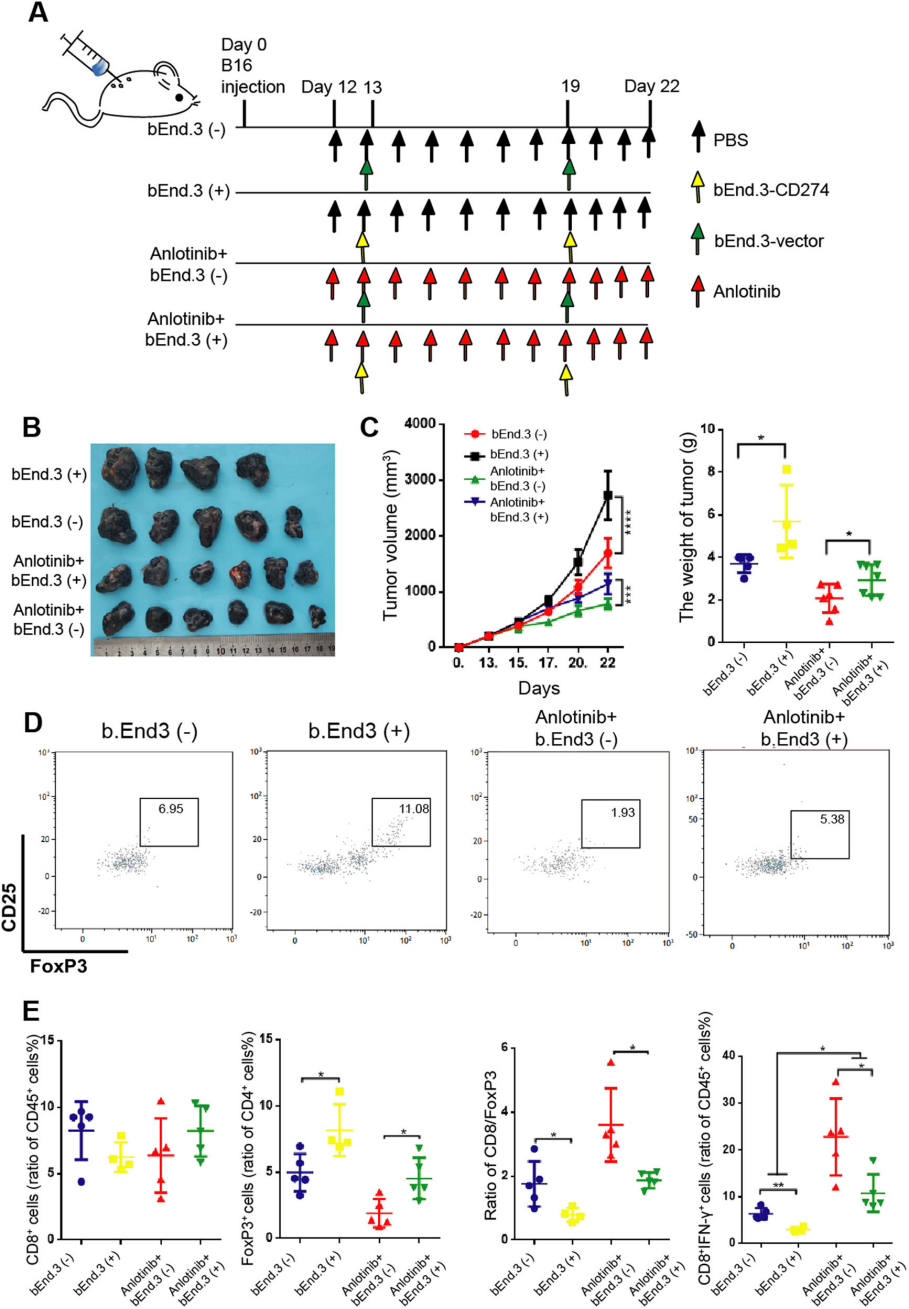
Endothelial PD-L1 expression affects the efficacy of anlotinib. **a** C57BL/6 mice were injected with 1 × 10^6^ B16 cells and tumors grew. On day 12, mice were divided into four groups (*n* = 8 per group), and treated with bEnd.3-vector, bEnd.3-CD274, PBS or anlotinib as shown. **b** Representative images of B16 tumor tissues treated as above indicated. **c** Left: tumor growth curve of the various treatment groups; right: Statistics of the weight of B16 tumors treated as indicated. **d** Representative flow images of CD4^+^ CD25^+^ FoxP3^+^ T cells taken from B16 tumors treated as indicated. **e** Statistics of the percentage of CD8^+^ T cells, CD8^+^ IFN-γ^+^ T cells, FoxP3^+^ T cells and ratio of CD8/FoxP3 in the tumors. Data are mean ± SD. *P < 0.05, **P < 0.01, ***P < 0.001, ****P < 0.0001.

To further determine the effect of PD-L1 expression on VECs, CD274 (PD-L1)-overexpressing lentivirus vector was transduced to bEnd.3 cells to construct a CD274-bEnd.3 (+) cell line, which could stably overexpressing PD-L1. We injected bEnd.3 (−) or CD274-bEnd.3 (+) cells into tumor tissues separately, and found that the tumor tissues with bEnd.3 (+) group (injected intratumorally CD274-bEnd.3 (+)) had a faster growth rate compared to the bEnd.3 (−) group (injected intratumorally bEnd.3 (−)) (Fig. 7a–c). In addition, in order to confirm whether VEC-PD-L1 had a role on the efficacy of anlotinib, we injected bEnd.3 (−) or CD274-bEnd.3 (+) cells intratumorally into tumor tissues separately with anlotinib treatment. Interestingly, we found that the therapeutic effect of anlotinib was assuaged in the anlotinib+bEnd.3 (+) group, and the B16 tumor tissues almost had the similar growth rate with the bEnd.3 (−) group. What’s more, anlotinib showed a promising curative effect in the anlotinib+bEnd.3 (−) group compared with the bEnd.3 (−) group, both the volume and the weight of tumor tissues were significantly decreased (Fig. 7a–c).

To further analyze whether this immunosuppressive phenomenon was caused by VEC-PD-L1, we collected tumor tissues from mice for flow cytometry. We found that the number of FoxP3^+^ T cells was increased in bEnd.3 (+) group, whereas the ratio of CD8/FoxP3 was lower compared with bEnd.3 (−) group (Fig.7d, e). The number of FoxP3^+^ T cells was decreased with anlotinib treatment (Fig. 7d, e). Although there was no significant difference in the number of CD8^+^ T cells among different groups, we found the activity of CD8^+^ T cells was enhanced and the number of CD8^+^ IFN-γ^+^ cells was increased after treatment with anlotinib (Fig. 7e). In addition, our study has confirmed that CD274-bEnd.3 cells can promote the infiltration of FoxP3^+^ T cells, reduce the ratio of CD8/FoxP3, and inhibit the function of CD8^+^ T cells with anlotinib treatment (Fig. 7d, e). These findings indicate that VEC-PD-L1 can not only affect the immune balance in tumor tissues, but also play an important regulatory role in the therapeutic effect of anlotinib.

## Discussion

VECs have been considered as a source of nutrition for tumor. However, with the development of research, it has been found that abnormal vasculature in tumor microenvironment also combines with the abnormal pericytes, forming dysfunctional tumor blood vessels, which contribute to the formation of an immunosuppressive environment^1^. Some studies^23,24^ have confirmed the view that blood vessels are not only a nutrient supply channel in tumor microenvironment, but also one of the important roles in immune regulation. Several studies^7,25,26^ suggest that VECs expressing PD-L1 is involved in autoimmune disease such as ARDS in the lung or skin inflammation, etc., and is closely related to the development of Treg cells and the inactivation of CD8^+^ T cells. Most studies^7,8,26^ focus on the impact of the PD-L1 expression on tumor cells or autoimmune disease, but few focus on the status of PD-L1 expression on VECs and its role in the tumor microenvironment.

In this regard, our study has demonstrated that angiogenic growth factors (VEGFA) and hypoxia are closely related to the PD-L1 expression on VECs in tumor. This result suggests that abnormal tumor environment may promote the expression of PD-L1 on VECs. According to previous literature, the PD-L1 expression on stromal cells is closely related to clinical stage^27^. We also found that the PD-L1 expression on VECs was closely related to clinical stage. However, owing to both the clinical stage and whole stromal cells were only rough index, wherein we may not find the real role of VEC-PD-L1, we further analyzed the correlation between PD-L1 expression on VECs and TNM stage (T stage, N stage, and M stage). We did not find a significant relationship between high percentage of CD34^+^ PD-L1^+^ vessels and any clinical variables, but *P* value was closed to significant point (0.066) in T stage. This result suggests that PD-L1 expression on VECs may be related to the T stage in primary tumor rather than N or M stage. In addition, our study also found that the high expression of PD-L1 on VECs independently predicted a poor prognosis, but had no association with the PD-L1 expression on tumor cells. This observation suggests that PD-L1 expression on VECs may be a new prognostic factor in several tumors independently from the PD-L1 expression on tumor cells.

In our study, we found that bevacizumab promoted the PD-L1 expression on VECs. Based on the previous literatures^28,29^ and our previous research^30^, we considered that bevacizumab could activate a variety of alternative pathways, such as the FGFR- and TGF-β-signaling pathway when it effectively neutralizes VEGFA, thereby activating the AKT pathway and finally causing upregulation of PD-L1 expression on VECs. In our vitro experiment, we found that blocking a variety of pro-angiogenic receptors (such as PDGFR, VEGFR2, and FGFR) by anlotinib inhibited the phosphorylation of AKT and inhibited PD-L1 expression on VECs induced by bevacizumab. Probably owing to the above effect, we found that anlotinib reduced the increase of mortality, tumor volume, and weight in mice induced by bevacizumab; simultaneously, PD-L1 was highly expressed on VECs in bevacizumab group, whereas inhibited on VECs in anlotinib group. These results suggest that VEC-PD-L1 is closely related to the immune microenvironment of tumor tissues and the efficacy of antiangiogenesis therapy.

According to previous studies^4,18^, the ratio of CD8/FoxP3 is considered to be one of the markers of tumor immune microenvironment, and patients with a high ratio of CD8/FoxP3 always have a better prognosis. In this study, we found the PD-L1 expression on VECs also acted as an “immune barrier” to inhibit the infiltration of effector immune cells and affect the ratio of CD8/FoxP3.

CD8^+^ T cells around blood vessels are considered to be the immune cells that have not penetrated the immune barrier and infiltrated into tumor tissues. In order to evaluate the tumor microenvironment more realistically, the ratio of infiltration of CD8^+^ T cells (excluding the perivascular CD8^+^ T cells) and the FoxP3^+^ T cells was deeply analyzed. We discovered that anlotinib could successfully promote CD8^+^ T cells to break through the “immune barrier” of VEC-PD-L1, infiltrate tumor tissues and significantly increase the ratio of CD8/FoxP3 compared with control group. The occurrence of this phenomenon may be attributed to the downregulation of PD-L1 expression on VECs, which leads to more easily infiltration of CD8^+^T cells into tumor and the activation of those cells before they infiltrated into tumor tissues. Of course, the really regulation mechanism still need to be future studied. Whatever it is or not, our data indicate that PD-L1 expression on VECs may have an important role in antiangiogenic therapy combined with immunotherapy. These results also suggest that anlotinib can effectively reverse the immunosuppression phenomenon by inhibiting PD-L1 expression on VECs induced by single-target antiangiogenic drug.

In T-cell depletion experiments, we found that the efficacy of anlotinib was closely related to CD8^+^ T cells in tumor tissues. In addition, we found that when the endothelial cells overexpressing PD-L1 were injected into tumor tissues, the tumor immunosuppressive environment was further aggravated and the proportion of CD8/FoxP3 was declined. These results indicate that the PD-L1 expression on VECs can affect the growth of tumor tissues through the formation of immunosuppressive microenvironment. The efficacy of anlotinib was also inhibited after injection of bEnd.3 (+) cells, which reduced the ratio of CD8/FoxP3. These results again suggest that the efficacy of anlotinib is closely related to the PD-L1 expression on VECs.

**Fig. 8:**
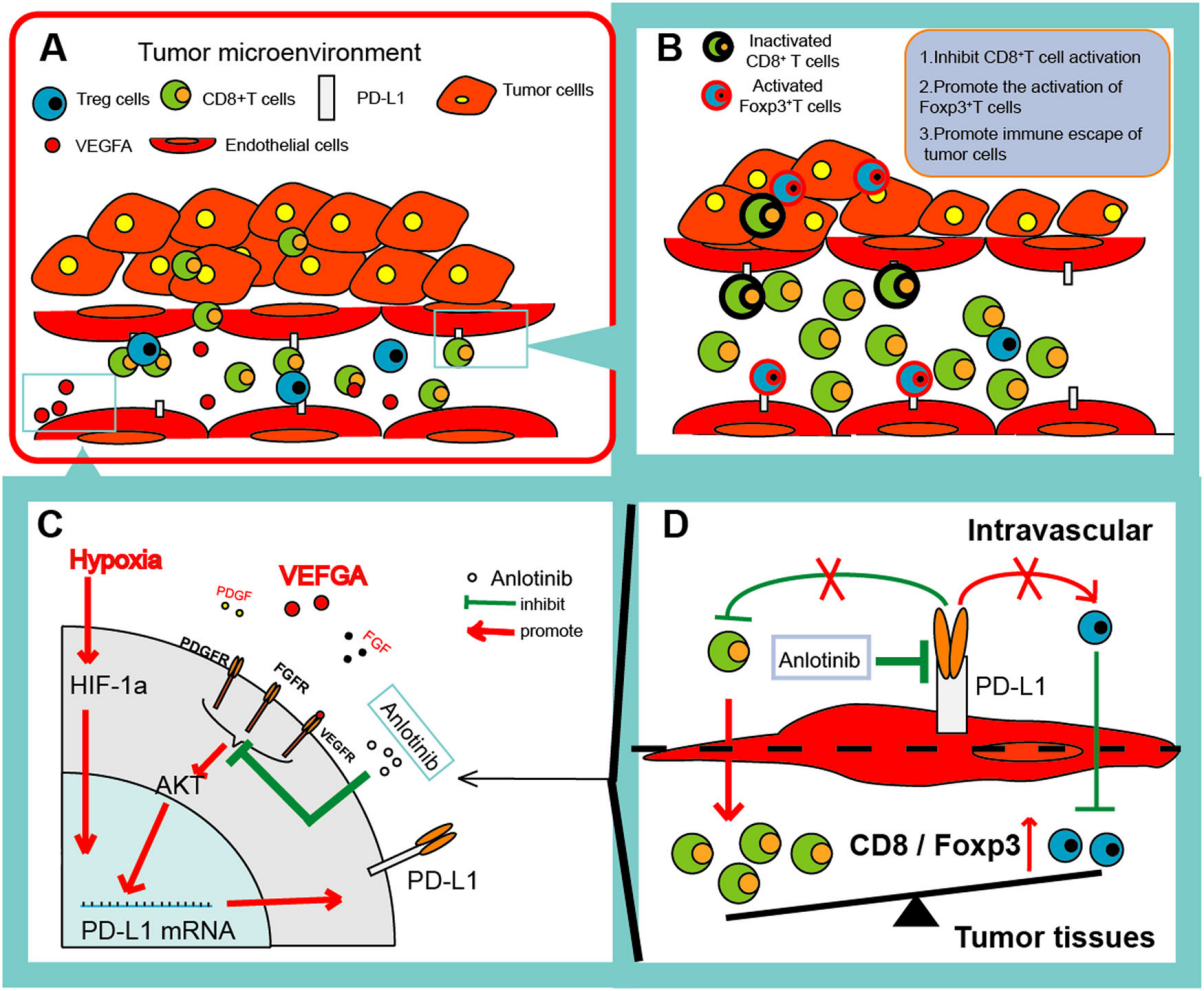
A schematic representation of effects of PD-L1 expression on VECs in tumor microenvironment. **a** Pattern diagram of tumor immune microenvironment. **b** PD-L1 highly expressed on VECs in tumor tissues inhibits activation of CD8^+^ T cells and promotes immune escape of tumors. **c** anlotinib inhibits the expression of PD-L1 on VECs through the AKT pathway. **d** anlotinib improved the ratio of CD8/FoxP3 and broke the immune barrier by inhibiting the VEC-PD-L1.

On the basis of these findings, a schematic representation for the effects of PD-L1 expression on VECs in tumor microenvironment was proposed (Fig. 8). Taken together, our findings indicate that the PD-L1 expression on VECs can realistically inhibit the immune-activation in microenvironment through promoting the aggregation of FoxP3^+^ T cells and inhibiting the function and infiltration of CD8^+^ T cells in tumor. Our study also indicates that anlotinib can regulate tumor immune microenvironment by inhibiting the expression of PD-L1 on VECs via AKT signaling pathway, which was not depend on its antiangiogenesis effects.

In summary, our experimental data demonstrate that VEC-PD-L1 has an important immunosuppressive role in the immune microenvironment and antiangiogenesis therapy, and illustrate the underlying mechanism of how anlotinib improved tumor immune microenvironment. The present study also provides a theoretical and experimental evidence for the combination of clinical anlotinib with immunotherapy.

## Supplementary information

Supplemental Table and Figure legends

Table S1

Fig S1

Fig S2

Fig S3
