# Supplementary material for: anlotinib alters tumor immune microenvironment by downregulating PD-L1 expression on vascular endothelial cells
Source: Cell Death Dis. 2020 May 4;11(5):309. doi: 10.1038/s41419-020-2511-3 (PMC7198575; doi:10.1038/s41419-020-2511-3)
Supplement: Supplementary file 1 — Supplemental Table and Figure legends [file 41419_2020_2511_MOESM1_ESM.doc]

**Supplemental Table and Figures legends**

**Table S1** The correlation of VEC-PD-L1 and the characters of patients with lung adenocarcinoma.

**Fig. S1:**

(A) Representative images of the expression of VEGFA and PD-L1 sections taken from subjects with vessels expressing either PD-L1^+^ or PD-L1^−^ in lung adenocarcinoma.

(B) Statistics of the number of microvascular, the expression of HIF-1α and VEGFA in tumors (n=41) from individuals that expressed either PD-L1^+^ or PD-L1^−^vessels.

(C) HUVECs were starved for 12 h, and treated with DMEM with 10% FBS, 50 ng/ml VEGFA or the supernatant of A549 cells (CM) for 24 h in hypoxia or normoxic conditions. The expression levels of PD-L1 were detected by western blot.

(D) HUVECs was treated with Anlotinib (0.1µM) or PBS for 24 h under different culture conditions. The expression levels of PD-L1 were detected by western blot.

(E) HUVECs was treated with Anlotinib (0.1µM), SU5408 (SU, 10uM), CP-673451 (CP, 4uM) and FIIN-2 (FI, 10uM) in different times as shown.

(F) HUVECs and bEnd.3 were treated with Anlotinib, Bevacizumab (10ug/ml) or both. In combined group, HUVECs were pre-incubated with Bevacizumab for 1 h, and treated with Anlotinib for 24 h or 3 h. Data are mean ± SD. *P < 0.05, **P < 0.01, ***P< 0.001, ****P< 0.0001, Student’s t test.

**Fig. S2:**

(A) Left: representative images of CD31 immunostaining (red), 4′,6-diamidino-2-phenylindole (DAPI) nuclear staining (blue) of B16 or MC38 tumors treated with Anlotinib or Bevacizumab; right: statistics of the area of vascular in different groups. (B) Tumor growth curve of the various treatment groups and statistics of the weight of MC38 tumor treated as indicated. (C) Left: Representative immunofluorescence images of CD31 (red) and PD-L1 (green) taken from the sections of B16 tumors; right: statistics of mean immunofluorescence intensity (MFI) of PD-L1 expression in tumor cells or CD31^+^ vessels. Data are mean ±SD. *P < 0.05, **P < 0.01, ***P< 0.001, ****P< 0.0001, Student’s t test.

**Fig. S3:**

(A) C57BL/6 mice were injected with 1**×**10^6^ B16-OVA cells and tumors grew. On day 11, mice were divided into 4 groups (n = 10 per group), and treated with αCD4, αCD8, Anlotinib, or both as shown.

(B) Left: tumor growth curve of the various treatment groups; right: Statistics of the weight of B16-OVA tumor treated as indicated. (C) Representative flow images of CD8^+^ or CD4^+^ T cells taken from the spleen of mice treated as indicated. Data are mean ±SD. *P < 0.05, **P < 0.01, ***P< 0.001, ****P< 0.0001, Student’s t test.
