# Supplementary material for: anlotinib alters tumor immune microenvironment by downregulating PD-L1 expression on vascular endothelial cells
Source: Cell Death Dis. 2020 May 4;11(5):309. doi: 10.1038/s41419-020-2511-3 (PMC7198575; doi:10.1038/s41419-020-2511-3)
Supplement: Supplementary file 2 — Table S1 [file 41419_2020_2511_MOESM2_ESM.doc]

**Table S1 The correlation of VEC-PD-L1 and the characters of patients with lung adenocarcinoma.**

| **Characters** | **Low,n (%)** | **High,n (%)** | **p-value** |
| --- | --- | --- | --- |
| T factor |  |  | 0.066 |
| ≤ T2 | 50(94.3) | 44(83.0) |  |
| > T2 | 3(5.7) | 9(17.0) |  |
| N factor |  |  | 0.212 |
| N0 | 39(73.6) | 33(62.3) |  |
| ≥N0 | 14(26.4) | 20(37.7) |  |
| M factor |  |  | 0.767 |
| M0 | 47(88.7) | 46(86.8) |  |
| >M0 | 6(11.3) | 7(13.2) |  |
| Gender |  |  | 0.836 |
| Male | 29(54.7) | 28(52.8) |  |
| Female | 24(45.3) | 25(47.2) |  |
| Age |  |  | 0.695 |
| ＜60 | 31(58.5) | 29(54.7) |  |
| ≥ 60 | 22(41.5) | 24(45.3) |  |
| Smoking history |  |  | 0.331 |
| Yes | 28(52.8) | 23(43.4) |  |
| No | 25(47.2) | 30(56.6) |  |
| EGFR mutations |  |  | 0.431 |
| Yes | 20(37.7) | 28(52.8) |  |
| No | 33(62.3) | 25(47.2) |  |

Statistically significant p-values (p< 0.05) was show in **bold**
