# Supplementary figures and images for: anlotinib alters tumor immune microenvironment by downregulating PD-L1 expression on vascular endothelial cells
Source: Cell Death Dis. 2020 May 4;11(5):309. doi: 10.1038/s41419-020-2511-3 (PMC7198575; doi:10.1038/s41419-020-2511-3)

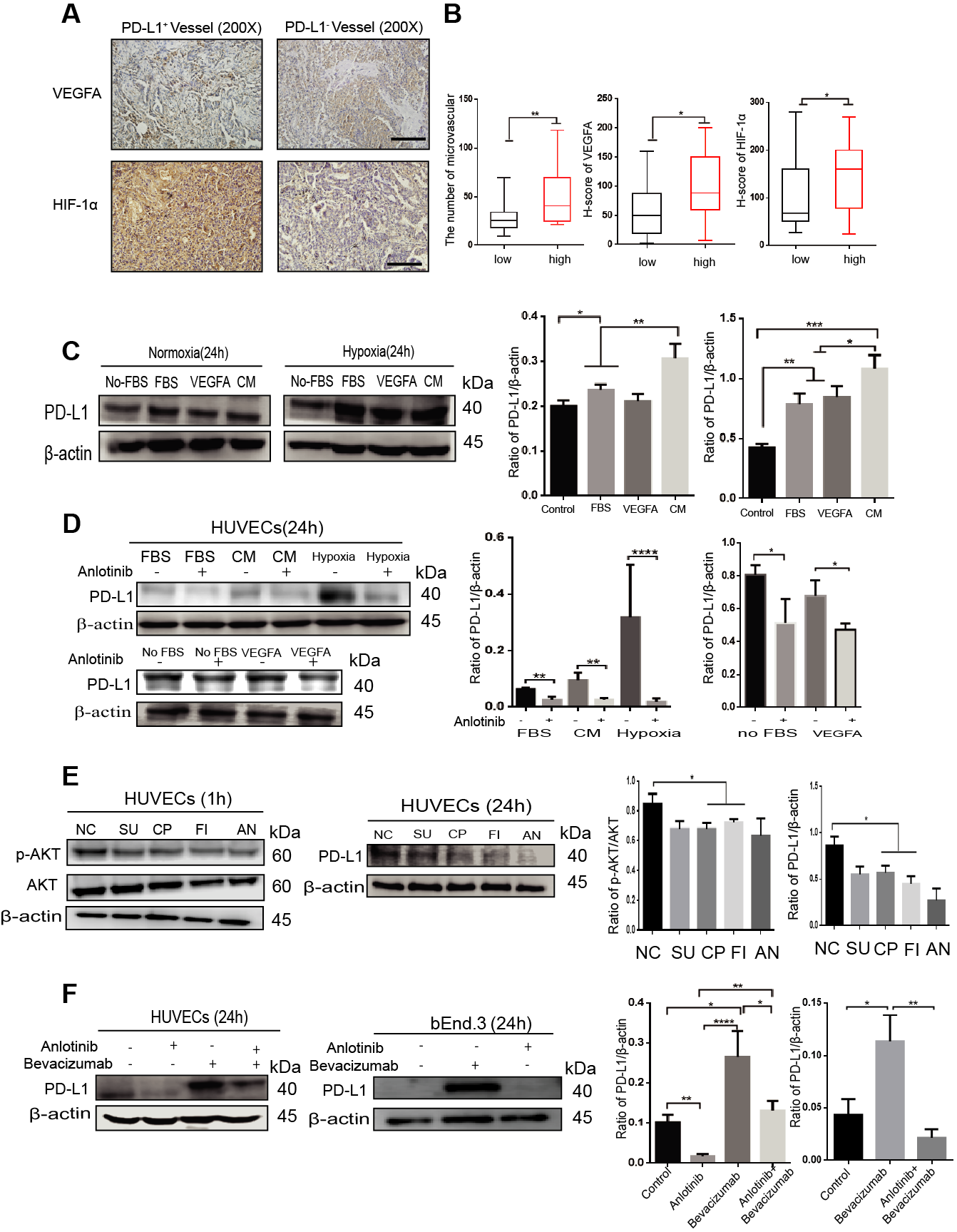

Supplement: Supplementary file 3 — Fig S1 [file 41419_2020_2511_MOESM3_ESM.png]

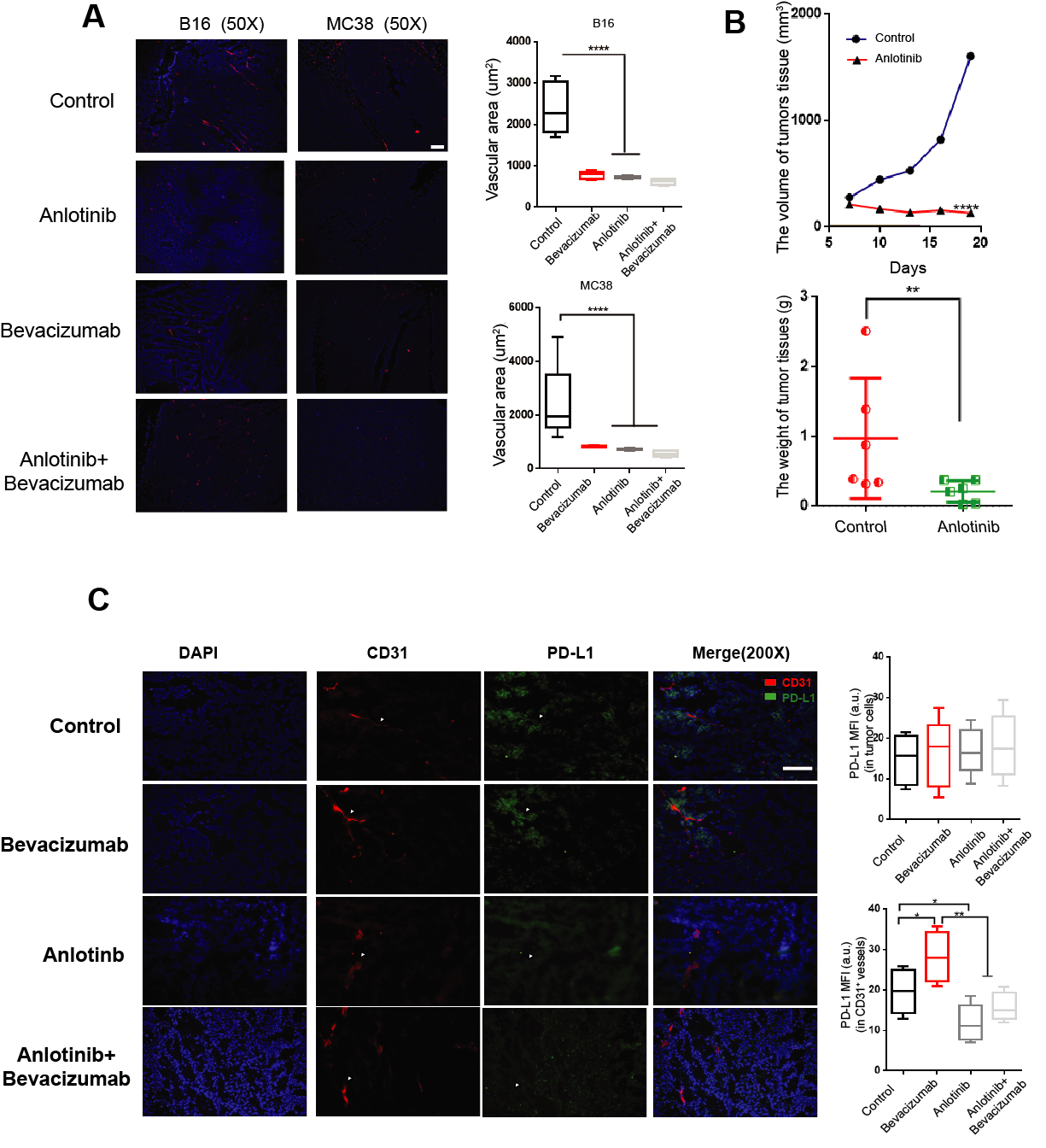

Supplement: Supplementary file 4 — Fig S2 [file 41419_2020_2511_MOESM4_ESM.png]

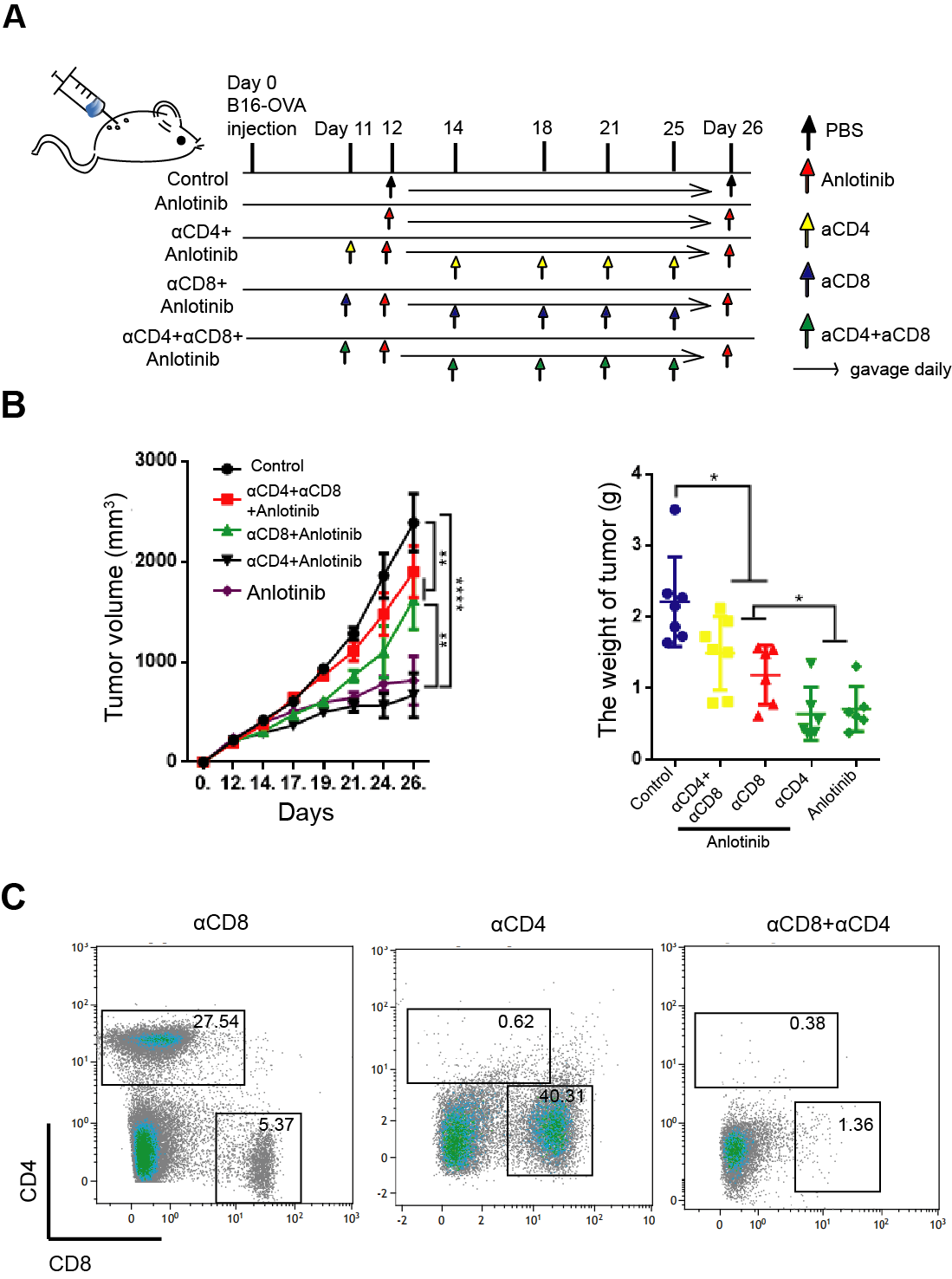

Supplement: Supplementary file 5 — Fig S3 [file 41419_2020_2511_MOESM5_ESM.png]
